# A Novel 2.5D Culture Platform to Investigate the Role of Stiffness Gradients on Adhesion-Independent Cell Migration

**DOI:** 10.1371/journal.pone.0110453

**Published:** 2014-10-13

**Authors:** Mark-Phillip Pebworth, Sabrina A. Cismas, Prashanth Asuri

**Affiliations:** Department of Bioengineering, Santa Clara University, Santa Clara, California, United States of America; Emory University/Georgia Insititute of Technology, United States of America

## Abstract

Current studies investigating the role of biophysical cues on cell migration focus on the use of culture platforms with static material parameters. However, migrating cells *in* *vivo* often encounter spatial variations in extracellular matrix stiffness. To better understand the effects of stiffness gradients on cell migration, we developed a 2.5D cell culture platform where cells are sandwiched between stiff tissue culture plastic and soft alginate hydrogel. Under these conditions, we observed migration of cells from the underlying stiff substrate into the alginate matrix. Observation of migration into alginate in the presence of integrin inhibition as well as qualitative microscopic analyses suggested an adhesion-independent cell migration mode. Observed migration was dependent on alginate matrix stiffness and the RhoA-ROCK-myosin-II pathway; inhibitors specifically targeting ROCK and myosin-II arrested cell migration. Collectively, these results demonstrate the utility of the 2.5D culture platform to advance our understanding of the effects of stiffness gradients and mechanotransductive signaling on adhesion-independent cell migration.

## Introduction

Until recently, investigations of mechanisms of cell migration focused on the use of two-dimensional (2D) tissue culture polystyrene (TCPS) surfaces, forcing cells to rely primarily on focal adhesions for forward traction. 2D cell migration begins with actin polymerization-mediated protrusion of the cell membrane, followed by the subsequent binding of transmembrane proteins such as integrins, and formation of focal adhesions at the cell front that anchor the cytoskeleton to the extracellular environment. [1]–[3] Myosin II then contracts the actin cytoskeleton to pull the cell along the direction of focal adhesion formation. [4]–[6] Such integrin-mediated formation of focal adhesions has been shown to regulate cell migration in 3D as well; [7]–[10] however, studies have also reported 3D cell migration in the absence of focal adhesions, which supports the existence of a second, amoeboid-like migration model. [11]–[13] In this form of migration, cells migrate via cytoskeletal rearrangements in a manner similar to amoebas to move through the dense network of interconnected pores in 3D. This amoeboid-like migration begins with the formation of large blebs, or rounded membrane protrusions, which flow and squeeze through fibers and pores and allow cell migration via purely mechanical means. [2], [14] Leukocytes have been shown to use this amoeboid-like form of migration to move rapidly through tissues of varying ECM composition and stiffness. [11], [12] Furthermore, studies have suggested that this mode of migration might also play a key role in cancer cell metastasis, which involves both the removal of adhesion points via ECM degradation as well as migration across transitions in microenvironmental stiffness [7], [15]–[18].

Currently, majority *in vitro* 3D cell culture models used for the assessment of leukocyte or cancer cell migration present a homogenous microenvironment devoid of elasticity changes that migrating cells experience *in* *vivo*. [19] Therefore, we developed a 2.5D culture platform where cells are placed at the interface between stiff TCPS and soft alginate to provide a more relevant model for studying the role of transitions in stiffness on cell migration. The alginate-based platform facilitated independent investigation of both matrix stiffness gradients and cell-matrix adhesions on migration. The roles of mechanotransductive pathways on cell migration in response to the stiffness gradients were also explored.

## Materials and Methods

### Cell culture

Standard mammalian cell culture practices were used for the maintenance of human HEK 293 and U87 glioblastoma cells (ATCC, Manassas, VA). Specifically, the cells were maintained in Dulbecco’s modified Eagle medium (DMEM) (Mediatech, Manassas, VA) supplemented with 15% fetal bovine serum (FBS) (Life Technologies, Carlsbad, CA), sodium pyruvate (Life Technologies), MEM non-essential amino acids (Life Technologies), and 1% penicillin-streptomycin (CellGro, Manassas, VA), and incubated at 37°C in a 5% CO_2_ humidified environment. Standard 60 and 100 mm cell culture plates (Greiner Bio-One, Monroe, CA) were used for passaging cells; the cells were grown to 60–80% confluency and subcultured at a 1∶4 ratio with 0.25% trypsin (CellGro, Manassas, VA).

### Alginate Preparation

High viscosity alginic acid sodium salt from brown algae (Sigma Aldrich, St. Louis, MO) was mixed in DI water to form a 3% w/v stock solution; the mixture was allowed to homogenize by magnetic stirring for 30 minutes, followed by overnight incubation at 37°C in a water bath. The alginate solutions were autoclaved at 121°C for 20 min for sterilization.

### Experimental Setup

**Figure 1:**
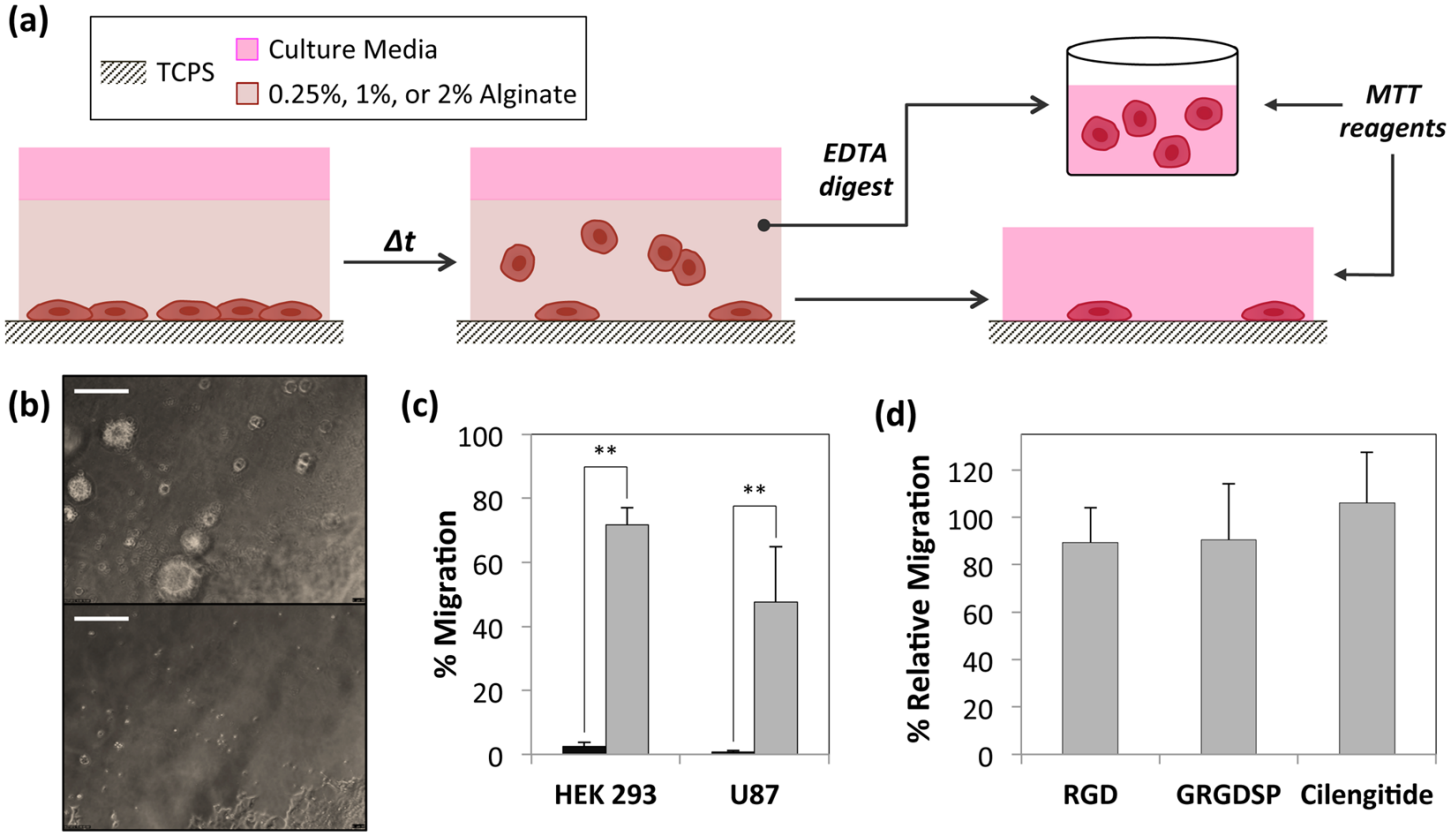
Cell migration under 2.5D culture conditions. (a) Schematic showing the experimental setup and procedure to investigate cell migration under 2.5D culture conditions. Cells sandwiched between TCPS and alginate were allowed to migrate over several days prior to subsequent alginate digestion. Cells in the alginate digest (migrated cells) and remaining attached cells were then quantified to calculate percent migration. (b) Representative pictures showing migrated HEK 293 cells into 0.5% alginate (top) and cells that remain attached to TCPS (bottom) on day 3. The scale bar depicts 200 µm. (c) Migration of HEK 293 and U87 cells into 0.5% alginate on day 6 (grey bars). Cell migration was also assayed immediately after alginate gelation (day 0, black bars) as a control. **P<0.01 for cell migration into 0.5% alginate on day 6 compared with control (i.e. cell migration immediately after alginate gelation), as determined by Student’s unpaired two-tailed t test. (d) Migration of HEK 293 cells into 0.5% alginate on day 3 in the presence of the integrin inhibitors – RGD, GRGDSP, and cilengitide relative to migration in the absence of the inhibitors. Error bars represent the standard deviation of three samples.

For the migration assays (as schematically shown in Figure 1a), cells were seeded into 48-well plates at a seeding density of ca. 12,000 cells per well (10–15% of the cell culture plate surface area). After 48 hours, the cell culture media was replaced with 300 µL of either 0.5 or 2% w/v solutions of alginate (diluted from the stock solution with media), followed by the addition of 300 µL of 100 mM CaCl_2_ solution to initiate gelation. After ca. 5 min, CaCl_2_ was replaced with 300 µL of fresh media; the cell culture media was replaced every 48 hours until the end of the experiment. In experiments involving inhibition of RhoA or Rac1 signaling and integrin binding, the inhibitors were added to the cell culture media immediately after alginate gelation (on day 0). Alginate concentration for the inhibitor experiments was set at 0.5% w/v. Integrin inhibitors and their respective concentrations (based on previous literature) were as follows: RGD that inhibits integrin binding to RGD motifs (Selleck Chemicals, Houston, TX) –200 µM, GRGDSP that inhibits integrin binding to fibronectin and vitronectin, adhesion proteins found in FBS (Sigma Aldrich, St. Louis, MO) –200 µM, and cilengitide that inhibits α_v_β_3_ and α_v_β_5_ (MedChem Express, Monmouth Junction, NJ) –5 µM. [20]–[23] Chemical inhibitor concentrations (based on previous literature) were as follows: Y-27632 that inhibits ROCK activity (Selleck Chemicals, Houston, TX) –16 µM, blebbistatin that inhibits myosin II ATPase activity (Cayman Chemical, Ann Arbor, MI) –5 µM, cytochalasin D that inhibits actin polymerization (Enzo Life Sciences, Farmingdale, NY) –1 µg/mL, and NSC23766 that inhibits Rac activation (EMD Biosciences, La Jolla, CA) –100 µM [24]–[26].

### Analysis of Cell Migration

At the appropriate time points, the media was replaced with an equivalent volume of 50 mM EDTA (BioRad, Hercules, CA) and incubated for 30 minutes at 37°C, 5% CO_2_. The digested alginate from each well was individually centrifuged for 2 minutes at 1,500 rpm, and the cells obtained were resuspended in 300 µL of 0.5 mg/mL MTT (ATCC) solution in DMEM. The MTT solution was also placed on the cell monolayer post alginate digest. These solutions were then incubated for 4 hours at 37°C before the addition of the detergent reagent (ATCC) for an overnight incubation. The final absorbance was read at 570 nm using a Tecan Infinite 200 PRO spectrophotometer (Durham, NC). The MTT absorbance reading for the digest was divided by the sum of the MTT readings for the digest and monolayer in order to find the percent migration for each well. Qualitative microscopic analyses were also performed on the 2.5D platform prior to alginate digestion. Gels were observed under the microscope to track the detachment of cells from the underlying monolayer on the TCPS and migrating into the alginate. These qualitative measurements were used as safeguards of the quantitative MTT-based measurements, as well as to prevent false positives or false negatives for migration.

## Results

### Cells at the TCPS-alginate interface migrate into alginate

In order to study the role of stiffness gradients on adhesion-independent cell migration, we cultured cells at the interface between stiff TCPS and soft alginate hydrogel layer (Figure 1a). For this we used alginate, an inherently bioinert biomaterial that lacks specific recognition sites for cell adhesion receptors [27] and therefore facilitates studies of cell migration that are independent of focal adhesions. Stiffness gradients were introduced by using alginate gels with elastic moduli ranging between 0.1–10 kPa that are significantly softer than TCPS (elastic modulus >1 GPa). Initial observations revealed cell migration of the model cell lines (HEK 293 and U87 glioblastoma) into 0.5% alginate with stiffness of ca. 300 Pa (Figure 1b and 1c and Figure S1). To confirm that focal adhesions did not play a significant role on the observed migration, we repeated the migration assays using the commercially available inhibitors (RGD, GRGDSP, and cilengitide) that have been shown to inhibit integrin-mediated adhesions and downstream cell fate decisions *in vitro*. [21], [28], [29] Neither of these inhibitors significantly impacted cell migration into alginate (Figure 1d); thus, these experiments confirmed the initial observations of adhesion-independent migration under the 2.5D culture conditions. Taken together, these results indicate that HEK 293 and U87 cells initially attached to TCPS move into alginate independent of integrin-mediated adhesions.

### Alginate matrix stiffness affects rate of cell migration

**Figure 2:**
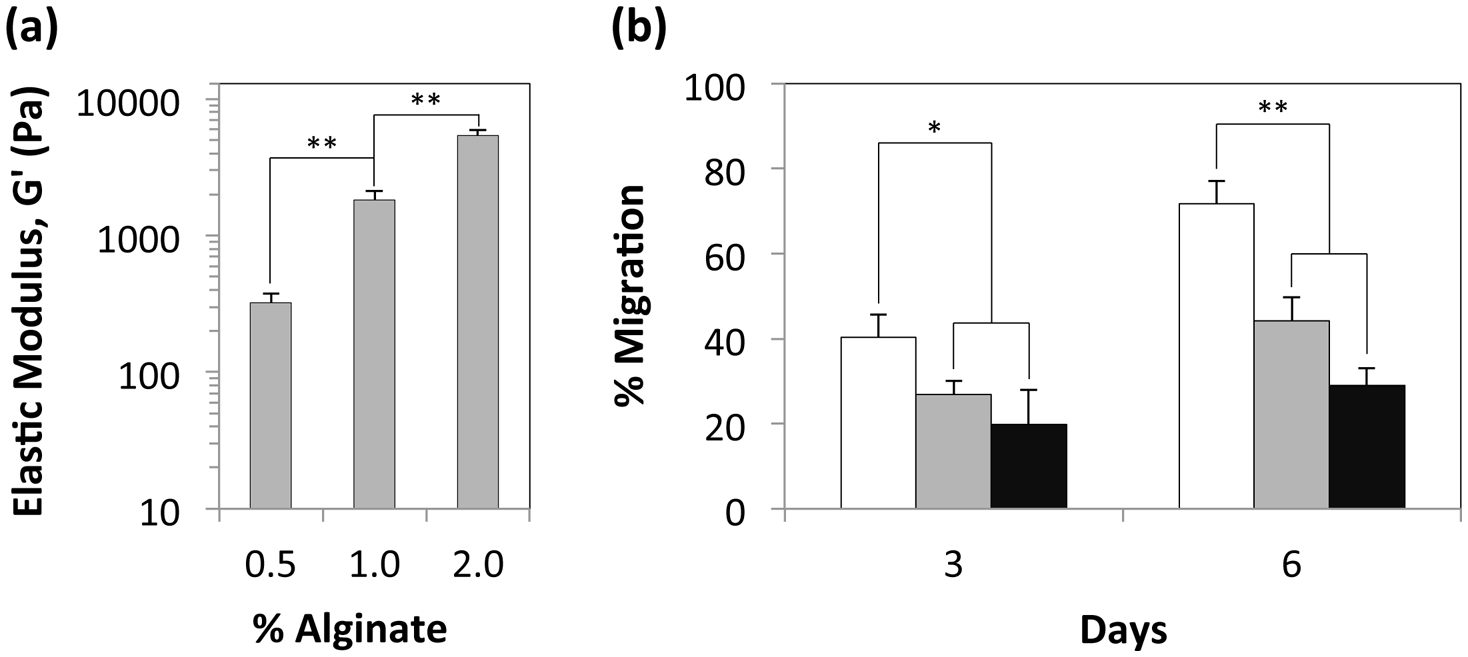
Influence of matrix stiffness on 2.5D cell migration. (a) Elastic modulus of 0.5%, 1%, and 2% alginate gelled using 100 mM CaCl_2_. **P<0.01 for elastic moduli of alginate hydrogels prepared using different concentrations of alginate, as determined by Student’s unpaired two-tailed t test. (b) HEK 293 cell migration into 0.5% alginate (white bars), 1% alginate (grey bars), and 2% alginate (black bars) at days 3 and 6. Statistical significance for cell migration into different concentrations of alginate was determined using Student’s unpaired, two-tailed t-test; *P<0.05 for migration into 0.5% alginate compared with migration in 1% and 2% alginate on day 3 and **P<0.01 for migration into 0.5% alginate compared with migration in 1% and 2% alginate on day 6. Error bars represent the standard deviation of three samples.

Next, we proceeded to study the effect of the overlying alginate matrix stiffness on 2.5D cell migration. We used three different concentrations of alginate –0.5%, 1%, and 2% for these analyses, as previous studies have shown significant differences in elastic moduli for alginate hydrogels for these concentration ranges. [30], [31] Rheological characterization revealed that the alginate hydrogel mechanical properties were clearly dependent on alginate concentration, and the elastic moduli for the different alginate concentrations were consistent with values previously reported in literature (Figure 2a). As seen in Figure 2b, we observed delayed migration of HEK 293s into 1% and 2% alginate relative to 0.5% alginate, suggesting the strong influence of matrix stiffness on 2.5D cell migration.

### Inhibition of RhoA-ROCK pathway inhibits cell migration

**Figure 3:**
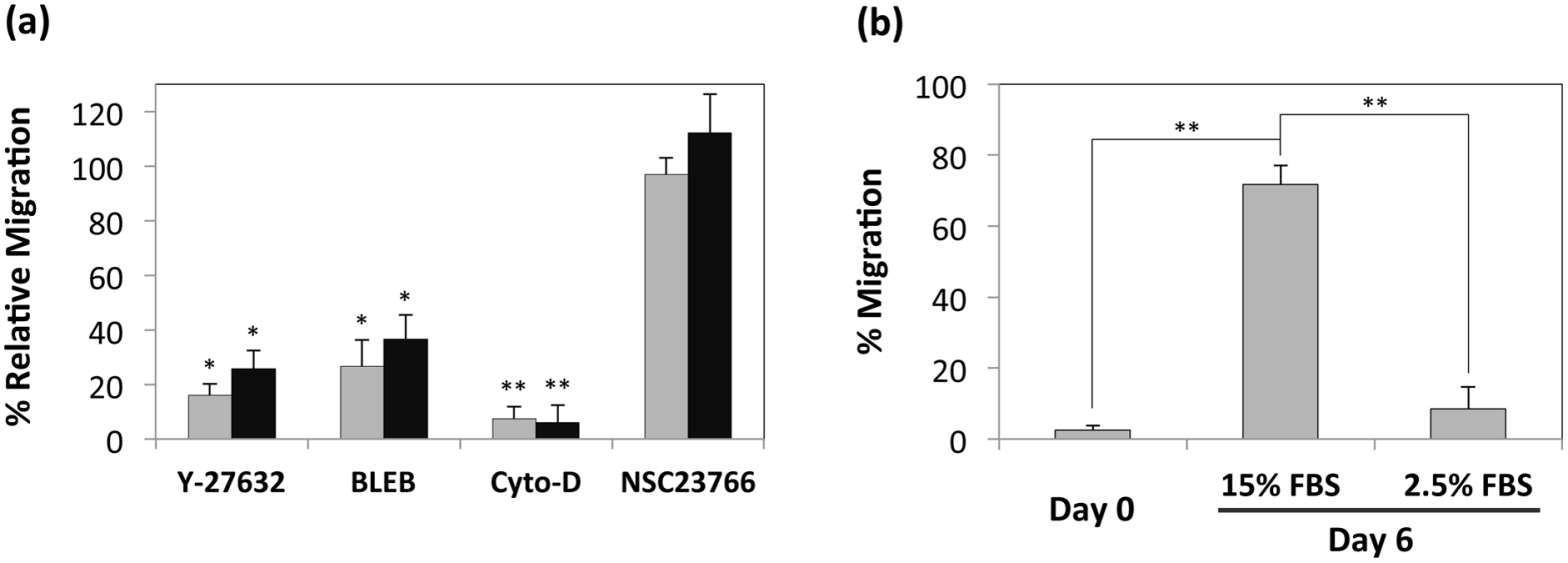
Role of mechanotransductive pathways on 2.5D cell migration. (a) Migration of HEK 293 (grey bars) and U87 cells (black bars) into 0.5% alginate on day 3 in the presence of small molecule inhibitors of ROCK – Y-27632, myosin activity – blebbistatin (BLEB), actin polymerization – cytochalasin D (Cyto-D), and Rac1– NSC23766 relative to migration in the absence of the inhibitors. Please note that the inhibitor vehicle DMSO did not impact cell migration. **P<0.01 for cell migration in presence of cytochalasin D and *P<0.05 for cell migration in presence of Y-27632 and blebbistatin, respectively compared with control (i.e. cell migration in the absence of the pathway inhibitors), as determined by Student’s unpaired two-tailed t test. (b) Migration of HEK 293 cells into 0.5% alginate in media containing either 15% or 2.5% FBS. Cell migration was also assayed immediately after alginate gelation (day 0) as a control. **P<0.01 for cell migration in media containing 15% FBS at day 6 compared with migration in media containing 2.5% FBS and control (i.e. cell migration immediately after alginate gelation), as determined by Student’s unpaired two-tailed t test. Error bars represent the standard deviation of three samples.

Having examined the role of alginate matrix biophysical properties on cell migration, we proceeded to obtain mechanistic insights behind the observed 2.5D cell migration. Previous investigations of 3D cell migration have indicated that RhoA activity, but not Rac1, is essential for alginate independent cell migration. [32]–[35] To test if RhoA signaling was involved in the cell migration observed in this study, we tested the role of small molecule inhibitors targeting various components of the RhoA pathway including ROCK (Y-27632), myosin II (blebbistatin), and actin (cytochalasin D) on cell migration into alginate. [36] Figure 3a shows inhibition of HEK 293 cell migration in the presence of these small molecule inhibitors; these results indicate the importance of the RhoA-ROCK-myosin II pathway on the observed 2.5D cell migration. Furthermore, inhibition of Rac1 using NSC23766 did not inhibit cell migration, suggesting that the Rac1 pathway was not implicated (Figure 3a). Furthermore, this trend was not unique to HEK 293s but was also seen for U87 glioblastoma cells, whose migration was also similarly dependent on the RhoA-ROCK (and not the Rac1) mechanotransductive pathway (Figure 3a). We conducted additional experiments that suggested the role of RhoA signaling in mediating 2.5D cell migration. The role of serum components on activation of the RhoA-ROCK pathway has been shown in previous studies; [37], [38] so we also performed the migration assays under varying concentrations of serum. We observed ca. 4-fold decrease in cell migration in media containing 2.5% serum relative to that in medium supplemented with 15% serum (Figure 3b).

## Discussion

Several studies have demonstrated the ability of stiffness gradients to regulate both 2D and 3D cell migration; however, these studies focus on the use of culture conditions that support adhesion dependent mechanisms of cell migration. [9], [39]–[43] For example, Tse *et al.* demonstrated that mesenchymal stem cells cultured on a collagen-coated polyacrylamide hydrogel presenting a stiffness gradient preferentially accumulate on stiffer hydrogel regions. [42] Likewise, Hadjipanayi *et al.* reported a similar observation in 3D; collagen matrices presenting a durotactic gradient guided cell migration to stiffer regions of the matrix. [41] While these studies contribute to our understanding of the relationships between stiffness gradients and lamellipodial mode of cell migration, it has been shown that cells can also migrate via alternate mechanisms *in* *vivo*. For example, leukocytes and cancer cells utilize adhesion-independent amoeboid-like cell migration mechanisms while transmigrating through the epithelium. [12], [17] And the effects of stiffness gradients on adhesion-independent cell migration have been relatively unexplored. Therefore, we sought to develop an *in* *vitro* platform that captured the effects of stiffness gradients on adhesion-independent cell migration. Cells were cultured between a stiff polystyrene substratum and soft hydrogel layer, which exposed the cells to a stiffness gradient. We chose alginate as the hydrogel matrix due to its lack of cell adhesion moieties. Previous research that used similar 2.5D culture platforms investigated the behavior of cells at interfaces of varying stiffnesses by sandwiching cells between collagen-coated TCPS and a thick layer of collagen. And these studies did not report cell migration into the soft collagen layer, possibly due to the strong presence of cell adhesion moieties. [9] Moreover, elastic modulus of alginate hydrogels can be controlled by changing the alginate concentration enabling facile investigation of matrix stiffness on cell fates and function. [30]–[31] Under these conditions, we observed a strong migration of cells into the alginate matrix within three days of culture at the TCPS-alginate interface. The observed migration was dependent on the stiffness of the alginate matrix, with enhanced rates of migration observed for soft alginate matrices. Finally, our mechanistic studies indicated that the observed migration was dependent on RhoA/ROCK activity. Our results are, therefore, in agreement with current investigations of various modes of cell migration that report switching between RhoA/ROCK-mediated bleb-like migration and Rac1-mediated lamellipodial migration [32], [33], [35], [44].

In conclusion, we have developed a novel culture platform that enables investigating the influence of stiffness gradients on adhesion-independent cell migration. Our data indicated the strong role of both matrix mechanical properties and mechanotransductive pathways in regulating the observed cell migration. However, further modifications to the experimental setup are warranted before the platform can be used to analyze migration mechanisms and pathways under conditions similar to those present *in* *vivo*. Specifically, we will focus on accurately mimicking specific stiffness gradients found *in* *vivo* by coating the TCPS with polymers displaying elastic moduli relevant to the biological frame of stiffness. In addition, further microscopy analyses in the form of fluorescent cell labeling and staining and confocal microscopy will advance our understanding of how cell morphology and receptor expression develops during 2.5D cell migration. Future experiments will also focus on establishing if the observations are general to other metastatic cancer cell lines and more importantly, if the platform can differentiate between metastatic and non-metastatic cancer cell lines. The results reported in this study and proposed experiments will be of interest to both basic and applied research. Our efforts will facilitate the development of optimal *in* *vitro* platforms that mimic *in* *vivo* conditions to study cancer cell migration and to discover therapeutic strategies against tumor cell motility and invasion [17].

## Supporting Information

Figure S1 **Qualitative microscopic analysis of 2.5D cell migration.** (a) Schematic of the microscopic analysis; pictures of cell migration were taken at various focal heights. Please note that the figure lines denoting the focal heights (1–6) are not to scale and are for representative purposes only. (b) Pictures of HEK 293 cells that remained attached to TCPS and those that migrated into alginate; numbers 1–6 correspond to pictures taken at various focal heights as represented in Figure S1a. The pictures were taken on day 3 prior to alginate digestion. The scale bar depicts 200 µm. Such qualitative analyses were also performed for various experimental conditions including different alginate matrix stiffnesses and the presence of inhibitors targeting RhoA-ROCK and Rac1 pathway.
(TIF)

## References

[1] Lauffenburger DA, Horwitz AF (1996) Cell migration: a physically integrated molecular process. Cell 84: 359–369. 8608589 10.1016/s0092-8674(00)81280-5

[2] Renkawitz J, Sixt M (2010) Mechanisms of force generation and force transmission during interstitial leukocyte migration. EMBO Rep 11: 744–750. 20865016 10.1038/embor.2010.147 PMC2948197

[3] Huttenlocher A, Horwitz AR (2011) Integrins in cell migration. Cold Spring Harb Perspect Biol 3: a005074. 21885598 10.1101/cshperspect.a005074 PMC3181029

[4] Yamaguchi H, Condeelis J (2007) Regulation of the actin cytoskeleton in cancer cell migration and invasion. Biochim Biophys Acta 1773: 642–652. 16926057 10.1016/j.bbamcr.2006.07.001 PMC4266238

[5] Vicente-Manzanares M, Ma X, Adelstein RS, Horwitz AR (2009) Non-muscle myosin II takes centre stage in cell adhesion and migration. Nat Rev Mol Cell Biol 10: 778–790. 19851336 10.1038/nrm2786 PMC2834236

[6] Fournier MF, Sauser R, Ambrosi D, Meister JJ, Verkhovsky AB (2010) Force transmission in migrating cells. J Cell Biol 188: 287–297. 20100912 10.1083/jcb.200906139 PMC2812525

[7] Even-Ram S, Yamada KM (2005) Cell migration in 3D matrix. Curr Opin Cell Biol 17: 524–532. 16112853 10.1016/j.ceb.2005.08.015

[8] Ulrich TA, Jain A, Tanner K, MacKay JL, Kumar S (2010) Probing cellular mechanobiology in three-dimensional culture with collagen-agarose matrices. Biomaterials 31: 1875–1884. 19926126 10.1016/j.biomaterials.2009.10.047

[9] Fraley SI, Feng Y, Krishnamurthy R, Kim DH, Celedon A, et al (2010) A distinctive role for focal adhesion proteins in three-dimensional cell motility. Nat Cell Biol 12: 598–604. 20473295 10.1038/ncb2062 PMC3116660

[10] Schmidt S, Friedl P (2010) Interstitial cell migration: integrin-dependent and alternative adhesion mechanisms. Cell Tissue Res 339: 83–92. 19921267 10.1007/s00441-009-0892-9 PMC2784868

[11] Friedl P, Borgmann S, Brocker EB (2001) Amoeboid leukocyte crawling through extracellular matrix: lessons from the Dictyostelium paradigm of cell movement. J Leukoc Biol 70: 491–509. 11590185

[12] Lammermann T, Bader BL, Monkley SJ, Worbs T, Wedlich-Soldner R, et al (2008) Rapid leukocyte migration by integrin-independent flowing and squeezing. Nature 453: 51–55. 18451854 10.1038/nature06887

[13] Renkawitz J, Schumann K, Weber M, Lammermann T, Pflicke H, et al (2009) Adaptive force transmission in amoeboid cell migration. Nat Cell Biol 11: 1438–1443. 19915557 10.1038/ncb1992

[14] Guck J, Lautenschlager F, Paschke S, Beil M (2010) Critical review: cellular mechanobiology and amoeboid migration. Integr Biol (Camb) 2: 575–583. 20871906 10.1039/c0ib00050g

[15] Zaman MH, Trapani LM, Sieminski AL, Mackellar D, Gong H, et al (2006) Migration of tumor cells in 3D matrices is governed by matrix stiffness along with cell-matrix adhesion and proteolysis. Proc Natl Acad Sci U S A 103: 10889–10894. 16832052 10.1073/pnas.0604460103 PMC1544144

[16] Phillips-Mason PJ, Craig SE, Brady-Kalnay SM (2011) Should I stay or should I go? Shedding of RPTPs in cancer cells switches signals from stabilizing cell-cell adhesion to driving cell migration. Cell Adh Migr 5: 298–305. 21785275 10.4161/cam.5.4.16970 PMC3210297

[17] van Zijl F, Krupitza G, Mikulits W (2011) Initial steps of metastasis: cell invasion and endothelial transmigration. Mutat Res 728: 23–34. 21605699 10.1016/j.mrrev.2011.05.002 PMC4028085

[18] Tozluoglu M, Tournier AL, Jenkins RP, Hooper S, Bates PA, et al (2013) Matrix geometry determines optimal cancer cell migration strategy and modulates response to interventions. Nat Cell Biol 15: 751–762. 23792690 10.1038/ncb2775

[19] Wu J, Mao Z, Tan H, Han L, Ren T, et al (2012) Gradient biomaterials and their influences on cell migration. Interface Focus 2: 337–355. 23741610 10.1098/rsfs.2011.0124 PMC3363018

[20] Shankar G, Davison I, Helfrich MH, Mason WT, Horton MA (1993) Integrin receptor-mediated mobilisation of intranuclear calcium in rat osteoclasts. J Cell Sci 105: 61–68. 7689577 10.1242/jcs.105.1.61

[21] Oliveira-Ferrer L, Hauschild J, Fiedler W, Bokemeyer C, Nippgen J, et al (2008) Cilengitide induces cellular detachment and apoptosis in endothelial and glioma cells mediated by inhibition of FAK/src/AKT pathway. J Exp Clin Cancer Res 27: 86. 19114005 10.1186/1756-9966-27-86 PMC2648308

[22] Amann K, Haas CS, Schussler J, Daniel C, Hartner A, et al (2012) Beneficial effects of integrin alphavbeta3-blocking RGD peptides in early but not late phase of experimental glomerulonephritis. Nephrol Dial Transplant 27: 1755–1768. 22049183 10.1093/ndt/gfr603

[23] Cheng NC, van Zandwijk N, Reid G (2014) Cilengitide inhibits attachment and invasion of malignant pleural mesothelioma cells through antagonism of integrins alphavbeta3 and alphavbeta5. PLoS One 9: e90374. 24595274 10.1371/journal.pone.0090374 PMC3940880

[24] Rubtsova SN, Kondratov RV, Kopnin PB, Chumakov PM, Kopnin BP, et al (1998) Disruption of actin microfilaments by cytochalasin D leads to activation of p53. FEBS Lett 430: 353–357. 9688570 10.1016/s0014-5793(98)00692-9

[25] Limouze J, Straight AF, Mitchison T, Sellers JR (2004) Specificity of blebbistatin, an inhibitor of myosin II. J Muscle Res Cell Motil 25: 337–341. 15548862 10.1007/s10974-004-6060-7

[26] Sivasubramaiyan K, Totey S, Bhat V, Totey SM, Deb K (2009) Y-27632 enhances differentiation of blastocyst like cystic human embryoid bodies to endocrinologically active trophoblast cells on a biomimetic platform. J Biomed Sci 16: 88. 19772618 10.1186/1423-0127-16-88 PMC2754421

[27] Rowley JA, Madlambayan G, Mooney DJ (1999) Alginate hydrogels as synthetic extracellular matrix materials. Biomaterials 20: 45–53. 9916770 10.1016/s0142-9612(98)00107-0

[28] Wu MH, Ustinova E, Granger HJ (2001) Integrin binding to fibronectin and vitronectin maintains the barrier function of isolated porcine coronary venules. J Physiol 532: 785–791. 11313446 10.1111/j.1469-7793.2001.0785e.x PMC2278579

[29] Wilisch-Neumann A, Kliese N, Pachow D, Schneider T, Warnke JP, et al (2013) The integrin inhibitor cilengitide affects meningioma cell motility and invasion. Clin Cancer Res 19: 5402–5412. 23948974 10.1158/1078-0432.CCR-12-0299

[30] Nunamaker EA, Otto KJ, Kipke DR (2011) Investigation of the material properties of alginate for the development of hydrogel repair of dura mater. J Mech Behav Biomed Mater 4: 16–33. 21094477 10.1016/j.jmbbm.2010.08.006

[31] Banerjee A, Arha M, Choudhary S, Ashton RS, Bhatia SR, et al (2009) The influence of hydrogel modulus on the proliferation and differentiation of encapsulated neural stem cells. Biomaterials 30: 4695–4699. 19539367 10.1016/j.biomaterials.2009.05.050 PMC2743317

[32] Ridley AJ (2001) Rho GTPases and cell migration. J Cell Sci 114: 2713–2722. 11683406 10.1242/jcs.114.15.2713

[33] Sahai E, Marshall CJ (2003) Differing modes of tumour cell invasion have distinct requirements for Rho/ROCK signalling and extracellular proteolysis. Nat Cell Biol 5: 711–719. 12844144 10.1038/ncb1019

[34] Sanz-Moreno V, Gadea G, Ahn J, Paterson H, Marra P, et al (2008) Rac activation and inactivation control plasticity of tumor cell movement. Cell 135: 510–523. 18984162 10.1016/j.cell.2008.09.043

[35] Petrie RJ, Yamada KM (2012) At the leading edge of three-dimensional cell migration. J Cell Sci 125: 5917–5926. 23378019 10.1242/jcs.093732 PMC4067260

[36] Lessey EC, Guilluy C, Burridge K (2012) From mechanical force to RhoA activation. Biochemistry 51: 7420–7432. 22931484 10.1021/bi300758e PMC3567302

[37] van Nieuw Amerongen GP, Vermeer MA, van Hinsbergh VW (2000) Role of RhoA and Rho kinase in lysophosphatidic acid-induced endothelial barrier dysfunction. Arterioscler Thromb Vasc Biol 20: E127–133. 11116077 10.1161/01.atv.20.12.e127

[38] Liu HW, Halayko AJ, Fernandes DJ, Harmon GS, McCauley JA, et al (2003) The RhoA/Rho kinase pathway regulates nuclear localization of serum response factor. Am J Respir Cell Mol Biol 29: 39–47. 12600823 10.1165/rcmb.2002-0206OC

[39] Lo CM, Wang HB, Dembo M, Wang YL (2000) Cell movement is guided by the rigidity of the substrate. Biophys J 79: 144–152. 10866943 10.1016/S0006-3495(00)76279-5 PMC1300921

[40] Isenberg BC, Dimilla PA, Walker M, Kim S, Wong JY (2009) Vascular smooth muscle cell durotaxis depends on substrate stiffness gradient strength. Biophys J 97: 1313–1322. 19720019 10.1016/j.bpj.2009.06.021 PMC2749749

[41] Hadjipanayi E, Mudera V, Brown RA (2009) Guiding cell migration in 3D: a collagen matrix with graded directional stiffness. Cell Motil Cytoskeleton 66: 121–128. 19170223 10.1002/cm.20331

[42] Tse JR, Engler AJ (2011) Stiffness gradients mimicking in vivo tissue variation regulate mesenchymal stem cell fate. PLoS One 6: e15978. 21246050 10.1371/journal.pone.0015978 PMC3016411

[43] Vincent LG, Choi YS, Alonso-Latorre B, del Alamo JC, Engler AJ (2013) Mesenchymal stem cell durotaxis depends on substrate stiffness gradient strength. Biotechnol J 8: 472–484. 23390141 10.1002/biot.201200205 PMC3749305

[44] Tsygankova OM, Wang H, Meinkoth JL (2013) Tumor cell migration and invasion are enhanced by depletion of Rap1 GTPase-activating protein (Rap1GAP). J Biol Chem 288: 24636–24646. 23864657 10.1074/jbc.M113.464594 PMC3750161

