# Supplementary figures and images for: A Novel 2.5D Culture Platform to Investigate the Role of Stiffness Gradients on Adhesion-Independent Cell Migration
Source: PLoS One. 2014 Oct 13;9(10):e110453. doi: 10.1371/journal.pone.0110453 (PMC4195729; doi:10.1371/journal.pone.0110453)

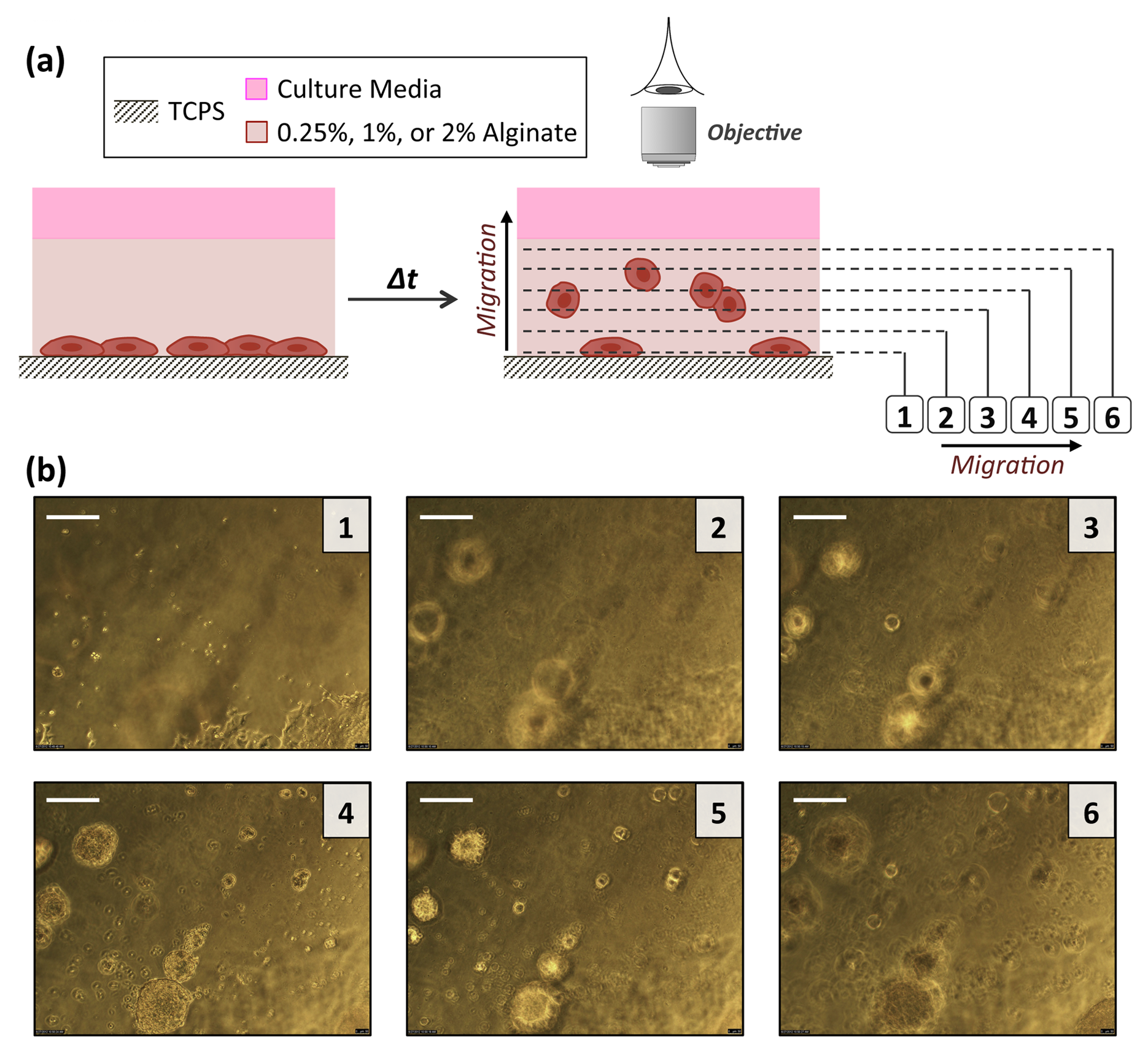

Supplement: Figure S1 — Qualitative microscopic analysis of 2.5D cell migration. (a) Schematic of the microscopic analysis; pictures of cell migration were taken at various focal heights. Please note that the figure lines denoting the focal heights (1–6) are not to scale and are for representative purposes only. (b) Pictures of HEK 293 cells that remained attached to TCPS and those that migrated into alginate; numbers 1–6 correspond to pictures taken at various focal heights as represented in Figure S1a. The pictures were taken on day 3 prior to alginate digestion. The scale bar depicts 200 µm. Such qualitative analyses were also performed for various experimental conditions including different alginate matrix stiffnesses and the presence of inhibitors targeting RhoA-ROCK and Rac1 pathway. (TIF) [file pone.0110453.s001.tif]
